# Anesthesia Experience for Open Gastrostomy with Ultrasound-Guided Erector Spinae Plane Block: A Case Report

**DOI:** 10.1155/2020/5413848

**Published:** 2020-03-26

**Authors:** Claude Thierry Bagaphou, Emanuele Piraccini, Lucia Norgiolini, Chiara Ciabucchi, Valentina Carsena, Luca P. Evoli, Domenico Pietro Santonastaso, Fabio Gori

**Affiliations:** ^1^Anesthesia and Intensive Care Unit, USL Umbria1 Ospedale di Città di Castello, Via L. Angelini 10, 06012 Città di Castello, PG, Italy; ^2^Anesthesia and Intensive Care Unit, Morgagni-PierantoniHospital, VialeForlanni 34, 47122 Forli, Italy; ^3^Department of General Surgery, USL Umbria1 Ospedale di Città di Castello, Via L. Angelini 10, 06012 Città di Castello, PG, Italy; ^4^Anesthesia and Intensive Care Unit, Azienda Romagna, M.Bufalini Hospital, 47521 Cesena, Italy; ^5^Section of Anesthesia, Intensive Care and Pain Medicine, Santa Maria Della Misericordia Hospital, Piazzale G. Menghini 1, 06129 Perugia, Italy

## Abstract

From the first description in 2016 till today, hundreds of studies have extensively presented Erector Spinae Plane block as an excellent perioperative analgesic technique especially in a multimodal pain management scenario. Only in few cases, this technique was used alone to provide surgical anesthesia.

## 1. Introduction

The erector spinae plane (ESP) block is a regional anesthesia technique described for the first time in 2016 by Forero et al. [1] for the management of thoracic neuropathic pain due to a rib fracture. In the last three years, interest in this technique has grown, and most publications present ESP block as an excellent technique for postoperative pain management [2].

The possibility and the indications to perform a surgical intervention with ESP block as the sole anesthetic technique has not still been fully discussed.

We report the use of a bilateral ESP block, with sedation, as sole anaesthesiological technique in a 50-year-old woman who required open gastrostomy surgery for esophageal stricture.

## 2. Case Report

The patient was informed and gave her authorization for possible publication of data and pictures.

The patient was a 50-year-old female, 55 kg, 167 cm, and we have assigned an American Society of Anesthesiologists physical status (ASA) of IV.

The patient presented with a history of postethylic liver cirrhosis, hepatorenal syndrome with cytolysis syndrome, cholestasis syndrome, and the platelet count was 80000/mm^3^.

She presented an El Ganzouri risk index of 5 (probable difficult intubation).

Furthermore, ten years ago, the patient was operated with resection of the glosso-palatine amygdale and emptying of the left lateral cervical region due to a heteroplasia of the left palatine tonsil, and five years ago, she underwent a placement of a tracheal stent for tracheal stenosis.

The patient asked, if possible, to undergo surgery without general anesthesia, and so, we opted for bilateral ultrasound-guided thoracic ESP block with sedation because a catheter or a thoracic epidural block would have been dangerous on a patient with liver cirrhosis, hepatorenal syndrome, and a platelet count of 80 000/mm^3^.

In the recovery room, the patient received standard heart rate, noninvasive blood pressure, and oxygen saturation monitoring and was placed in the sitting position.

50 mcg of intravenous fentanyl was administered before ESP block administration.

The puncture area and ultrasound probe (linear transducer 7–13 MHz) were prepared in a sterile manner; the ultrasound probe, positioned into a longitudinal orientation to obtain a parasagittal view, was placed over the midline of the back at the desired level and then slowly moved laterally until the transverse process was visible as a hyperechoic structure, with acoustic shadowing below it.

We viewed trapezius muscle, rhomboid muscle, and the erector spinae muscle (ESM) over the transverse process.

The puncture was performed with a 22-G × 50mm needle (Pajunk, GmbH, Geisingen, Germany), using the “in-plane” technique; the correct position of the needle on the transverse process was confirmed by the injection of 1 ml of 0.9% saline solution and the visualization of linear fluid spreading deep to the ESM.

So, we administered 7 ml of levobupivacaine 0.5%. at T6 level and 7 ml at T8 level on the left, and the same volume of the same solution was injected at T6 and T8 level on right side. We injected the local anesthetic on two levels to ensure good cranio-caudal spread and therefore better anesthesiological coverage.

As described in the literature, the diffusion of the local anesthetic is not only between the muscle fascia towards the lateral anterior region of the thorax, but also spreads towards the paravertebral space. The ESM extends all along the spine, as a consequence, so the solution spreads in cranial and caudal direction, and thus, several dermatomes can be anesthetized [3, 4]. 25 min later in the operating room, before sedation, the test with pin prick and cold sensation with alcohol-soaked sponge showed the diffusion of local anesthetic from T3 level up to T11 level [4].

Sedation with target controlled infusion (TCI Alartis PK, CareFusion) of propofol was administered with an initial target concentration of 4mcg/ml, shifting then to a maintenance dose of 2 mcg/ml.

**Figure 1:**
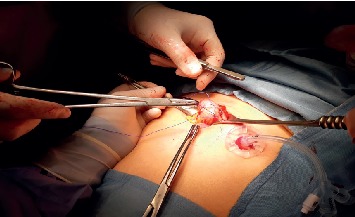
Surgical incision.

An epigastric skin incision along the centre line was performed. The abdominal cavity was cautiously accessed, exposing the front wall of the gastric body (Figure 1). Surgical gastrostomy was then prepared according to La Fontaine, with placement of a 20F feeding tube.

Surgery was completed in 35 minutes, and the patient did not complain of pain and maintained spontaneous respiration and no opioids were required.

Acetaminophen 1 gr IV was administered at the end of surgery and then every eight hours, and postoperative pain was assessed using the NRS Pain Scale at time 0, at the end of surgery, and at 2, 6, 12, and 24hours after surgery. Ketorolac 30 mg, prescribed as rescue analgesic, was never required.

Pain never exceeded 1 on the NRS Pain Scale. No postoperative nausea and vomiting were reported.

The patient was discharged 30hours after surgery.

## 3. Discussion

Most publications reported in the current literature present ESP block as an excellent technique for regional anesthesia with several indications for thoracic, breast, abdominal, gynecological, neck, and even for hip surgery. It is an easy and safe technique that–combined with multimode analgesia–reduces the use of morphine to treat postsurgery pain [2, 5, 6]. Over 242 cases reviewed in the literature and a bibliographical research carried out on 140 studies confirm that single-shot ESP block or with catheter is an excellent analgesic technique that may become a future valid alternative to epidural analgesia both in thorax and abdominal surgery[7, 8]. However, only few cases report about ESP block as sole anesthetic technique, and the use of ESP block for gastrostomy has still not been described. Since the first report about its use for the management of chronic pain after a thoracotomy [1], the ESP block has become a revolutionary analgesic technique, but the question is whether it can be consistently indicated as a valid anesthetic technique. Our work reports the case of a patient with a esophageal stricture who underwent open gastrostomy with ESP block successfully, associated with a sedation with spontaneous breathing, as the sole anesthetic technique. This result confirms the case report described by Hu et al. who performed ESP block as an anesthesiological technique for nonintubation video-assisted thoracoscopic surgery [9]. There are also 3 reports of ESP block being used as a surgical anesthesia method in minor surgeries; Tulgar et al. published that ESP block could be used as the main anesthetic method for peri-paravertebral area surgical procedure [10] and for the management of ileostomy closure under ESP block at 8th thoracic vertebral level due to risk of general anesthesia in the patient. High volume and concentration of 0.5% bupivacaine and 2% lidocaine was applied with successful result [11]. Balaban et al. reported 3 cases of minor surgery at thoracic region under ultrasound-guided ESP block [12].

The action mechanism of the ESP block is not completely known yet, but most authors have demonstrated that by injecting the local anesthetic under the fascia of the ESM (over the transverse process), the ventral and dorsal spinal sensitive branches are blocked [13].

The ESP block has been described in the last few years as an excellent analgesic technique within the multimode approach for the management of postsurgery pain in several surgical indications. In the literature, the term ESP block is often associated to analgesia, reduction of the consumption of morphine, and postsurgical pain.

With our work, we want to underline that, in selected cases for minor surgery, the ESP block can also be used as the only anesthetic technique.

There is a need for randomized studies on multiple patients to establish the real utility of ESP in this typology of surgery and to establish the right dose of local anesthetic to be administered.
